# An Ionization-Based Aerosol Sensor and Its Performance Study

**DOI:** 10.3390/s24175600

**Published:** 2024-08-29

**Authors:** Yong Zhang, Chunqi Wang, Liang Xie, Yuqi Peng, Ruizhe Wang

**Affiliations:** 1 School of Instrument Science and Technology, Xi’an Jiaotong University, Xi’an 710049, China; 2 State Key Laboratory of Electrical Insulation and Power Equipment, Xi’an Jiaotong University, Xi’an 710049, China; 3 School of Electrical Engineering, Xi’an Jiaotong University, Xi’an 710049, China; 3122104001@stu.xjtu.edu.cn (C.W.); 3121304002@stu.xjtu.edu.cn (L.X.); pengyuki2001@stu.xjtu.edu.cn (Y.P.); 15191447504@163.com (R.W.)

**Keywords:** silicon micron column, ionization type, MEMS, aerosol sensor

## Abstract

In recent years, with the rapid development of new energy vehicles, the safety issues of lithium-ion batteries have attracted attentions from all sectors of society. Research has found that during the thermal runaway process of lithium-ion batteries, aerosol emissions usually occur earlier than other gases. Accurate and timely measurement of these aerosol concentrations can help to warn the power battery pack fires. However, existing aerosol sensors are unable to meet the requirements of real-time monitoring and high precision. This article proposes an ionization mechanism based aerosol sensor that works at principles of field emission, field charging and gas discharge, and investigates its static and dynamic response characteristics. The sensor is manufactured and assembled using Microelectro Mechanical Systems processing technology. The sensor exhibits superior performances in terms of range, sensitivity, nonlinearity, repeatability, response time, and other aspects. The study provides a new solution for current aerosol detection with great potential for application.

## 1. Introduction

With the rapid expansion of the electric vehicle market, customers’ expectations for key performance indicators such as battery range and battery energy storage capacity continue to rise. Lithium-ion batteries have become the mainstream power source for electric vehicles due to their advantages, such as high energy density, high specific energy, high voltage, long lifespan, and low self-discharge [1]. According to research by NASA [2], lithium-ion batteries release a large amount of aerosols when thermal runaway occurs. These aerosol particles are mostly smaller than 5 μm, with the majority being smaller than 2.5 μm, and form stable aerosols inside the battery pack. Aerosol refers to a gaseous dispersion system composed of solid or liquid particles suspended in a gas medium, with particle sizes typically ranging from 0.001 to 100 μm [3,4,5]. The study by S.R. Cummings et al. [6] found that during the thermal runaway process of lithium-ion batteries, aerosol emissions usually occur earlier than CO and H_2_, providing a possible technical approach for early warning of the thermal runaway. Szymon Jakubiak [7] used an ionizing aerosol sensor designed by the standard radioactive source Am-241, which continuously emits strong radiation to ionize the background gas, and produces positive and negative ions. The sensor detects aerosol concentration by measuring the potential changes on the electrode caused by the collisions between the aerosol particles and ions. Shimadzu (Japan, Kyoto) [8] has designed an ionizing smoke sensor with needle-shaped electrodes installed inside. After being powered on, it detects the smoke by ionizing the background gas through the corona discharge that occurred at the electrode tip. Zhang Chao from Tsinghua University [9] estimated the average relative errors of aerosol concentration and particle size measured by a cutting-edge corona discharge based aerosol sensor to be 12.2% and 13.5%, respectively, but the aerosol sensor only responds well to small particle sizes. Maik Bertke [10] designed a size-selective sampling and removal system for airborne nanoparticles (NPs) using a silicon resonant microcantilever mass balance combined with a miniaturized electrostatic precipitator (ESP), but quantitative concentration tests were not performed.

For aerosol concentration detection, widely used systems are bulky, time-consuming, or expensive to maintain. Over the past decade, sensor technology has developed towards the following features: portable, inexpensive, and suitable for monitoring real-time measurements. PM monitoring systems with micro-size can be easily accessed with the development of nano or micro-scale systems [11]. The study is based on the theories of field emission, field charging, and gas discharge [12,13], and utilizes microelectro-mechanical system (MEMS) technology to design a highly sensitive and accurate ionized aerosol sensor, which is suitable for aerosol detection in power battery packs. Due to its small size and fast response speed, the sensor is suitable for installation and operation in limited spaces on electric vehicles. It can detect the concentration of aerosol particles inside the battery pack and warn the thermal runaway. Compared with traditional aerosol particle sensors [14,15,16], it has a smaller volume, higher sensitivity, and faster response time. The work provides an innovative method for the safety inspection of power battery packs, with potential for engineering applications.

## 2. Materials and Methods

### 2.1. The Basic Structure of the Sensor

Field emission materials in ionized aerosol sensors have the following characteristics: good physical and chemical properties; not easily affected by external electric fields; ability to meet the demand for high current density emissions; lower operating voltage; a certain degree of resistance to particle bombardment and antioxidant properties; low cost; simple manufacturing process; and good repeatability. Therefore, silicon was chosen as the material for the cathode [17].

**Figure 1:**
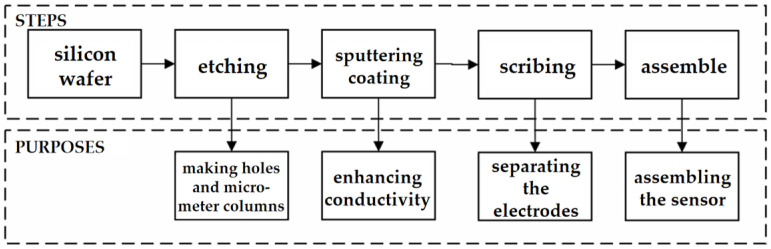
The fabrication process of the sensor.

The fabrication process of the sensor is shown in Figure 1. The sensor was based on a single crystal silicon wafer, and holes were drilled on the cathode and the extracting electrode through an etching process, as well as grooves dug on the collecting electrode. Next, deep silicon etching technology [18] was used to produce silicon micro-columns. The silicon micro-column structures used in this sensor have a height of 100 μm and a diameter of 20 μm, that is, the depth-to-width ratio of the micro-column is up to 5:1. The distance between the centers of adjacent silicon micro-columns is 50 μm. Deep Reaction Ion Etching (DRIE) technology ensures the accuracy of silicon micro-column etching and solves the difficulties in the high-aspect-ratio manufacturing process. DRIE technology manufactures high aspect ratio silicon microstructures by alternating thin layer coverage and silicon substrate etching steps. In addition, the DRIE process exhibits lower etching sidewall roughness in high aspect ratio etching processes, which helps to improve the performance of microstructures. Afterwards, sputtering coating technology was used to deposit a Cr–Au conductive thin film on the micro-columns. The main purpose of Cr thin film is to promote adhesion between silicon wafers and Au layers. The Au layer coating not only improves the conductivity of the silicon wafer surface, but also has good corrosion resistance. At last, a whole block of silicon wafer was cut into the cathode, the extracting electrode and the collecting electrode.

**Figure 2:**
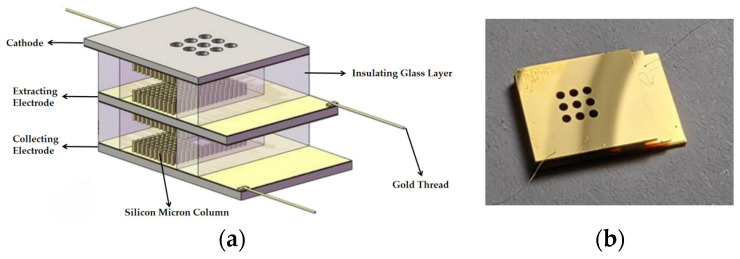
Schematic of the aerosol sensor, (**a**) The four sided micro-column based on three electrode structures and (**b**) the physical photo of the sensor.

**Figure 3:**
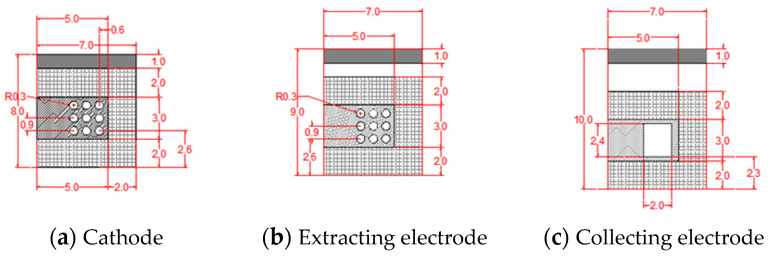
Dimension of the electrodes (mm).

The sensor consists of a cathode, an extracting electrode, and a collecting electrode (Figure 2a), which are arranged in order from top to bottom. To control the electrode spacing and ensure the insulation performance, a quartz glass film with a thickness of 230 µm was placed between the electrodes. Finally, the gold threads [19] were fixed at the edge of the electrodes through wire bonding technology [20,21] to achieve the power supply and response current collection. Thesensor is shown in Figure 3.

Nine diffusion holes were constructed on the cathode silicon wafer, and a silicon micrometer column array was covered on the side facing the extracting electrode. The diffusion hole serves as a channel for aerosols to enter the sensor while reducing electrode damage caused by excessive positive ions bombarding the cathode micro-column. The size of the extracting electrode is also covered with a silicon micro-column array on both sides, which also has nine diffusion holes. The main function of the extracting electrode is to guide the positively charged aerosol particles generated by collision with positive ion clouds so that they can successfully pass through the holes under diffusion and finally reach the collecting electrode under electric field driving. Grooves were engraved at the corresponding positions of the collecting electrode diffusion holes to adsorb charged particles that reach the collecting electrode. The outer side of the groove was also covered with a silicon micro-column array with the same structures as the cathode. The dimensions of the electrodes are shown in Figure 3 and Table 1.

When the sensor is in operation, the cathode emits high-energy electrons that collide between the electrodes to produce positive ions. The cathode material is affected by the impact of positive ions, causing damage to the microcolumns above it and affecting the sensitivity and working life of the aerosol sensor [22]. To address this challenge, the preparation of structurally stable and particle-resistant micro/nanostructures has become crucial [23]. In this study, while maintaining an appropriate aspect ratio, the column diameter and spacing were increased, and a silicon micro-column structure with a depth-to-width ratio of 5:1 was selected as the cathode emission material. For the improvement plan of the micrometer structure, the stability and durability of the sensor have been significantly enhanced while improving the reliability and consistency of manufacturing. In addition, the micro- column structure between the extracting electrode and the cathode can enhance the electric field strength and effectively increase the number of high-energy electrons emitted by the cathode. By preparing silicon micro-columns around the collection tank, the effective collection area was expanded, and the collection capacity for charged particles was improved. This innovative design is crucial for the stability of the overall performance of the sensor while reducing the risk of fracture and short circuits caused by inter-column collisions. These improvements help to reduce the risk of damage caused by particle collisions while improving the collection efficiency and overall performance stability of the sensor.

### 2.2. Principle of Aerosol Sensor Detection

**Figure 4:**
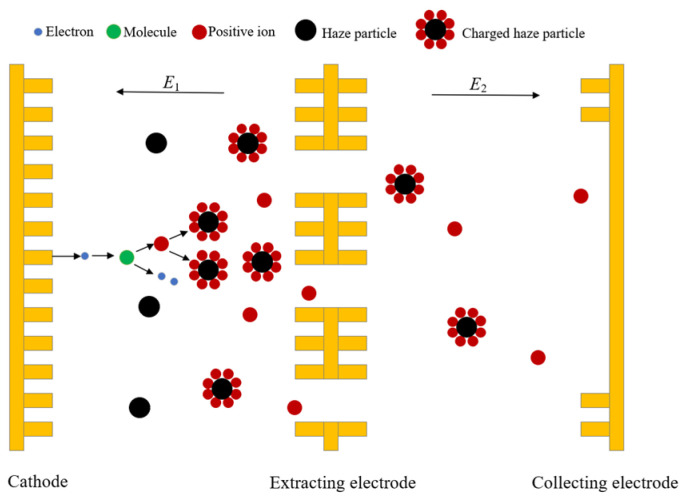
The detection principle of the aerosol sensor.

As shown in Figure 4, under external excitation, the tip of the micro-column on the cathode plate emits high-energy electrons through the field emission phenomenon [24]. Under the strong electric field formed between the cathode and the extracting electrode, electrons gain sufficient energy to overcome the surface work of the cathode and move toward the extracting electrode. At the same time, electrons collide and ionize with background gas molecules, producing a large number of positive ions [25]. These positive ions will collide with aerosol particles entering the discharge space between the cathode and the outlet, causing the particles to be charged. Under the interaction between aerosol particles and positive ions, charged particles and gas positive ions pass through the extraction hole together by the concentration gradient driving force and move along the direction of the electric field to the collection electrode. Under the strong electric field between the discharge and collection electrodes, charged particles eventually move to the collection electrode and form the collecting current, *I*_c_. By measuring the magnitude *I*_c_ value, it is possible to detect the concentration of aerosol particles.

The reaction formulas for electrons colliding with background gas molecules and producing a large number of positive ions are as follows:

e + N_2_ → N_2_^+^ + e + e (1)

e + N_2_ → N_2_(A^3^Σ_u_^+^) + e (2)

e + N_2_ → N_2_(a’^1^Σ_u_^−^) + e (3)

N_2_(A^3^Σ_u_^+^) + N_2_(a’^1^Σ_u_^−^) → e + N_2_ + N_2_^+^ (4)

N_2_(A^3^Σ_u_^+^) + N_2_(a’^1^Σ_u_^−^) → e + N_4_^+^ (5)

N_2_(a’^1^Σ_u_^−^) + N_2_(a’^1^Σ_u_^−^) → e + N_2_ + N_2_^+^ (6)

N_2_(a’^1^Σ_u_^−^) + N_2_(a’^1^Σ_u_^−^) → e + N_4_^+^ (7)

N_2_^+^ + N_2_ + N_2_ → N_4_^+^+ N_2_ (8)

Particle charging refers to the process in which particles are charged when they pass through the electric field between the sensor electrodes. According to the charging mechanism, it can be divided into two categories: electric field charging and diffusion charging [26]. The size of particulate matter has a significant impact on its charging process. For particles with a size of 0.5 μm, electric field charging is the main charging process. The amount of charge obtained by particles entering a charged electric field is shown in the equation below [27]:

(9) $$Q=A_{\varepsilon }\;\frac{\varepsilon }{\varepsilon +2}\;E_{0}\;D^{2}\;(\frac{1}{1+\tau /t})$$

where A_ε_—electric field velocity constant (F·m^−1^); ε—relative dielectric constant; *D*—particle diameter (m); *E*_0_—field strength (V·m^−1^); *τ*—Charge time constant (s); *t*—time (s). After particles enter the electric field, the charging process proceeds rapidly and takes an extremely short time. For particulate matter, its charging time constant is 10^−3^ s [28].

The formula for collecting current and charge is as follows:

(10) $$I_{c}=\frac{dQ}{dt}$$

where *Q*—total charge of charged particles (C); *I*_c_—collecting current (A).

The high-energy electrons emitted by the cathode collide and ionize with the background gas under the action of an electric field, producing a large number of positive ions. The total current depends on the original discharge current *I*_0_ in the electrode gap near the micro-columns and electrode separation *d*, as follows:

*I* = *I*_0_ e*^αd^* (11)

where *I*_0_—original discharge current (A); *d*—electrode separation (m); α—the first ionization coefficient of gas.

Based on the multivariate relationship between the first ionization coefficient of gas α, maximum field strength *E*, and the background gas partial pressure *P* [29], we have the following formula:

*α* = *AP*exp(−*BP*/*E*) (12)

where *E*—electric field strength at the tip of the micro-columns on the cathode (V/m); *P*—pressure (Pa); and *A* and *B*—constants related to gas type, pressure, and field ratio without unit.

Combining the electric field strength formula:

(13) $$E=\frac{U}{d}$$

where *U*—pole to pole voltage of the sensor (V); *d*—spacing between the electrodes (m), it indicates that the electric field strength *E* is the highest in the tip area of the micro-columns between the sensor electrodes due to the nonuniformity electric field distribution near the column tips with micrometer curvature radius.

The ideal gas law exhibits: *PV* = *n*R*T*, where *P* represents pressure (Pa), *V* represents volume (m^3^), *n* represents the amount of substance (mol), R is the universal gas constant without unit, and *T* represents temperature (K). According to Formula (12), it can be concluded that the first ionization coefficient α is related to the amount of background gas substances. Formula (11) indicates that the collection current generated by gas discharge remains unchanged until aerosol particles enter the sensor. The gas-positive ions generated by the collision between sensor poles are the source of charge for aerosol particles entering the sensor. We assume that the total charge of charged aerosol particles falling on the collecting electrode is *Q*_1_, and the charge of particles retained in the interpole region is *Q*_2_. Combining the Formula (10), the collecting current of the sensor is:

(14) $$I_{c}=I_{0}\;e^{\alpha d}+\frac{dQ_{1}}{dt}-\frac{dQ_{2}}{dt}$$

According to Equation (9), it can be inferred that the maximum charge occurs when aerosol particles enter the tip area of the micrometer column between the electrodes of the sensor. The electric field strength remains unchanged here, and only the concentration and particle size of the particles affect the charge of the aerosol sensor. Adding it to Equation (14) demonstrates that the output current *I*_c_ varies with the concentration of aerosol particles, which indicates that the ionizing aerosol sensor in this study is able to detect the concentration of aerosol particles.

### 2.3. Experimental System for the Sensor

**Figure 5:**
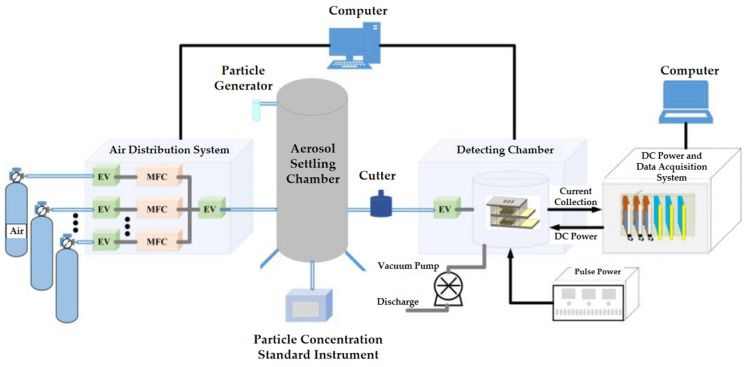
Schematic diagram of the experimental system.

In order to deepen the research on the function of aerosol sensors, it is crucial to accurately control the concentration of aerosols and measure the accuracy of the collector current. Therefore, a specialized aerosol detection experimental system was constructed, as shown in Figure 5. The core components of the system include a particle generation system, an experimental detection room, a data collection system, and a piece of power equipment. The particulate matter generator system includes a gas distribution system and a particulate matter generator. The mass flow controller (MFC) of the gas distribution system is connected to the corresponding gas source bottle to provide appropriate pressure for the particulate matter generator and can set the background gas for aerosol particles. The particulate matter generator can generate aerosol particles of various diameters. The experimental detection room is used to place aerosol sensors and is equipped with a vacuum pump inside, which can effectively extract residual gases and aerosol particles. The particle concentration standard instrument is TSI8534 (TSI, Beijing, China), which provides the standard concentration of aerosol for the experiment. The data acquisition system is responsible for accurately measuring the current output of the sensor. The pulse voltage required by the sensor is provided by a high-frequency single pulse power supply [30].

#### 2.3.1. Aerosol Particle Generator System

The particle generator in the aerosol particle generator system can be filled with silica particles of different particle size ranges and matched with a particle size cutter to simulate aerosols of various particle size ranges. In order to adjust the aerosol concentration, the background air cylinder is first connected to the high-flow mass flow channel of the gas distribution system. Then, with the help of a computer-aided automatic gas distribution system, the flow rate of the flow meter is adjusted, and an electromagnetic valve (EV) is opened to send the gas into the particulate matter generator. Finally, the gas transports aerosol particles to the gas pipeline, and the aerosol particles enter the particle settling chamber together with the gas. Through this system, aerosol particles with different diameters and concentrations can be effectively configured.

#### 2.3.2. Aerosol Particle Sensor Detection Room

The aerosol particle sensor detection room is an experimental piece of equipment designed specifically for studying the performance of aerosol sensors, with four independent chambers in the detection room. Before conducting aerosol detection, the sensor needs to be fixed in the chamber, and the wiring of the three electrodes should be led out to the data acquisition system through shielded wires. Afterwards, the chamber was closed, its sealing ensured, and the air inside the chamber was extracted through a vacuum pump to eliminate the interference of residual gases and aerosol particles, which provided a clean reference environment for subsequent aerosol concentration measurement. After the pressure in the chamber reaches the preset vacuum level, the aerosol particle generator system introduces the configured aerosol into the detection chamber. After aerosols are flowed into the chamber under the pressure difference, the sensor began to detect aerosol particles in real-time and the performance of the sensor was studied under various concentration and particle size conditions.

#### 2.3.3. Power Supply System of the Sensor

A high-frequency single pulse source, YS9000D, provides the pulse power supply to the extracting electrode, and its detailed technical parameters are shown in Table 2. NI PXI-4132 provides a constant DC voltage to the collecting electrode of the sensor.

#### 2.3.4. Data Acquisition System of the Sensor

The seven-and-a-half-digit multimeter module PXI-4071, produced by National Instruments of the United States, can achieve high-precision and high-resolution current measurement, and provide data support for sensor performance research. In the application of sensor output current, PXI-4071 can achieve a wide measurement range from 10 fA to 3 A, accurately measuring the sensor output current. The PXI-4071 module is placed in the NI PXI-1044 chassis and can be connected to the remote controller module to achieve communication with the computer. On this basis, with the help of the control program written in LabVIEW 2022 software, users can conveniently monitor and record the changes in the output current of the sensor in real-time, thereby studying the performance of the sensor under different working conditions is feasible.

## 3. Results

### 3.1. Optimization Experiment of Pulse Excitation Parameters

The tip of the micro-column of the sensor experiences electron collisions with charged particles, which are affected by the electric field. The characteristics of pulse excitation help to reduce the impact on the cathode structure, which maintains the reliability of the sensor. This phenomenon effectively reduces the impact of positive ions on micro-columns, prolongs the service life of sensors, and maintains consistency [31]. In the low-frequency range, increasing the pulse excitation frequency will increase the number of collisions between electrons and gas molecules, and further enhance the ionization effect. However, with the frequency further increased, the movement of electrons is limited by the rapidly changing direction of the electric field, which makes sufficient energy for collision ionization obtained difficult. This indicates that there exists an optimal pulse excitation frequency at a specific pole spacing. The duty cycle will affect the duration and interval of the ionization process, thereby affecting the ionization and collection processes of aerosol particles. If the duty cycle were large, the duration of the ionization process would be longer within one cycle, which could ionize more aerosol particles and generate a larger collection current. However, if the duty cycle were too large, the interval time of the ionization process would be shortened, which might lead to continuous ionization, cause changes in the distribution of the electric field in space, affect the movement and distribution of aerosol particles in the electric field, and thus affect the collection current. Therefore, at a specific pole spacing, there exists not only an optimal frequency but also a duty cycle selection for the pulse excitation, which requires careful consideration of various factors such as the aerosol particle size, the aerosol concentration, and the sensor design.

In the experiment, dry air was employed as the background gas. The experimental conditions are shown in Table 3, and the adjusted parameters are the frequency and duty cycle of the pulse excitation. By detecting the collecting current of the sensor, the influence of pulse frequency and duty cycle on the sensor is studied and the combination of pulse frequency and duty cycle is optimized to improve performance of the sensor.

**Figure 6:**
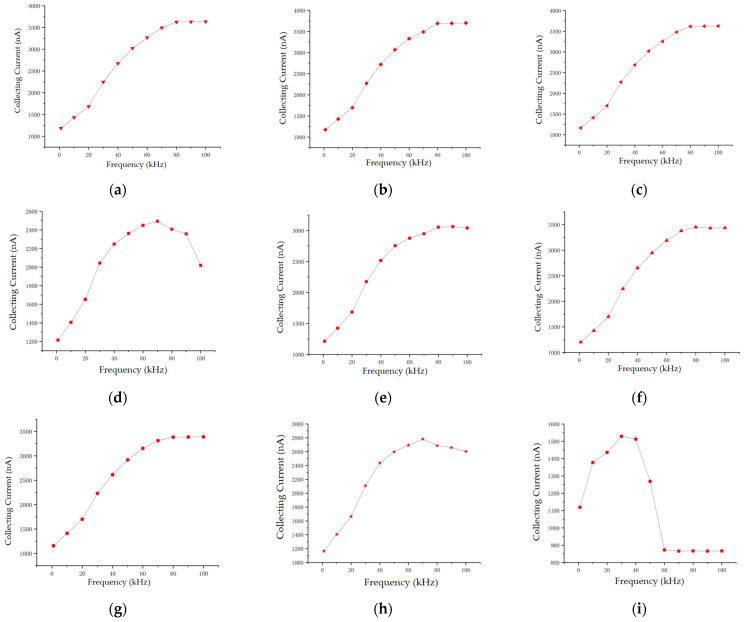
Characteristic curves of *I*_c_ versus pulse frequency under different duty cycles: (**a**) 10%; (**b**) 20%; (**c**) 30%; (**d**) 40%; (**e**) 50%; (**f**) 60%; (**g**) 70%; (**h**) 80%; and (**i**) 90%.

Figure 6 shows the sensitivity characteristic curves between the collecting current of the sensor and the pulse excitation parameters. It suggests that the collecting current *I*_c_ generally shows an upward trend with the increasing of the pulse frequency. Within the duty cycle range from 20 to 70%, *I*_c_ tends to stabilize at the pulse frequency of 80 kHz. At the duty cycle range of 10%, 80%, and 90%, with the pulse frequency increasing *I*_c_ first increases and then decreases, and a peak of the *I*_c_ curve appears at approximately 80 kHz. Thus in order to ensure the largest collection current and highest sensitivity of the sensor, the pulse frequency is optimized to be 80 kHz.

**Figure 7:**
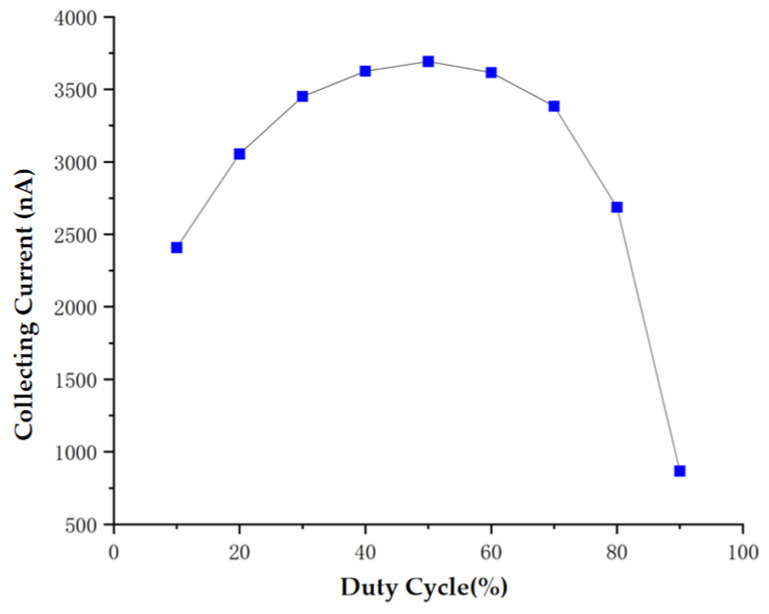
Characteristic curves of Ic vesus duty cycle under 80 kHz condition.

At a fixed pulse frequency of 80 kHz, the effect of pulse width on the collection current of the sensor was observed. As shown in Figure 7, the largest collection current of the sensor corresponds to 50% duty cycle. Based on the principle that ensures a large output current of the sensor, 50% duty cycle is selected as the optimal experimental excitation parameter. In this case, the sensor can more accurately capture and measure changes in aerosol particles, which significantly improves the performance and reliability of the sensor.

In summary, the optimal external conditions for sensor detection are shown in Table 4.

### 3.2. Static and Dynamic Response of the Sensor

#### 3.2.1. Static Response of the Sensor

**Figure 8:**
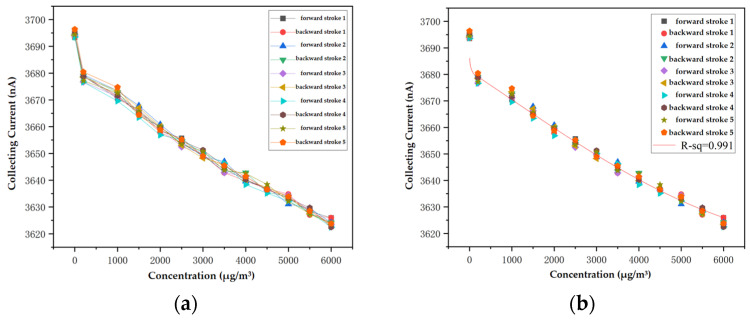
Response of the sensor to aerosols, (**a**) Collecting current versus concentration of aerosol and (**b**) Nonlinear fitting output result of the sensor.

This section aims to study the sensitivity characteristics of sensors to aerosols under optimal pulse excitation conditions and explore the indicators of sensor repeatability, hysteresis, and nonlinearity. This experiment selected an aerosol concentration range of 0–6000 μg/m^3^ and set 13 concentration calibration points. According to the pulse excitation conditions in Table 3, sensitive characteristic data were obtained by measuring the concentration changes of 2.5 μm diameter aerosols in five different strokes of the sensor. The corresponding output characteristic curve of the sensor is shown in Figure 8. It can be seen that as the aerosol concentration increases, the collecting current of the sensor descends continuously, and at the low aerosol concentration the collecting current decreases rapidly.

By using nonlinear fitting methods to process data from multiple sensor curves, an input–output model of the sensor can be established. The selected nonlinear fitting model is:

*I*_c_ = *a*_0_ + *a*_1_ × *φ*_2.5μm_ + *a*_2_ × *φ*^2^_2.5μm_ + *a*_3_ × *φ*^2.5^_2.5μm_ + *a*_4_ × exp(−*φ*_2.5μm_) (15)

where *I*_c_—collection current (A), *φ*_2.5μm_—concentration of the 2.5 μm diameter aerosols (μg/m^3^), *a*_0_, *a*_1_, *a*_2_, *a*_3_, *a*_4_—undetermined constants. The concentration on the exponential function is normalized to 1 µg/m^3^.

The fitting results of the sensor obtained through calculation are shown in Table 5.

The partial static characteristic indicators of the sensor obtained through calculation are shown in Table 6.

The minimum detection limit is the minimum concentration that can cause a change in the sensor output.

Uncertainty is a quantitative expression of the possible deviation in the measurement results. The formula for type A uncertainty is:

(16) $$\mu_{A}=t\cdot \sqrt{\frac{\sum_{i=1}^{n}\;{\left(x_{i}-\overset{\_}{x}\right)}^{2}}{n(n-1)}}$$

where *n*—number of the measurements; *t*—testing factor, with a confidence probability of 95%; *x_i_*—measurement data; and $\overset{\_}{x}$—average value of the measurements.

#### 3.2.2. Dynamic Response of the Sensor

In order to study the dynamic response characteristics of the sensor, a sensor with a separation of 30 μm was selected, and experimental conditions were set according to Table 3. By observing and recording the changes in the collected current *I*_c_ of the sensors over time, the dynamic response behavior of the sensors at different concentration conditions was demonstrated, and the performance of the sensors in particle detection was reflected.

**Figure 9:**
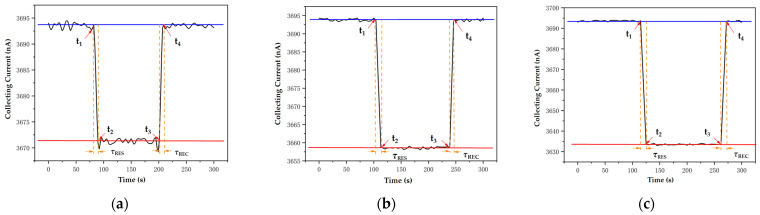
Dynamic response curve of 2.5 μm aerosol: (**a**) 1000 μg/m^3^; (**b**) 3000 μg/m^3^; (**c**) 6000 μg/m^3^.

Through experiments, the dynamic response curves of aerosols were obtained at concentrations of 1000 μg/m^3^, 3000 μg/m^3^, and 6000 μg/m^3^. As shown in Figure 9, it can be observed that after introducing aerosols into the detection chamber, the collecting current *I*_c_ of the sensor changes rapidly and tends to stabilize quickly. Similarly, after clearing the indoor aerosol, the collecting current of the sensor also rebounds.

In this study, the dynamic response characteristics of the sensor are determined by the response time *τ*_RES_ and recovery time *τ*_REC_ to be measured. Response time *τ*_RES_ is defined as the time required for the collection current *I*_c_ to increase from a stable value of 10% to 90% after the aerosol sample enters the detection room, which reflects the sensor’s adaptability to new inputs. The recovery time *τ*_REC_ is the time required to collect the current *I*_c_ from a stable value of 90% to 10% after reintroducing dry air, reflecting the speed at which the sensor recovers from its activated state to its initial state. These two indicators can reflect the rapid response and recovery ability of sensors to changes in aerosol concentration. Sensor response time *τ*_RES_ and recovery time *τ*_REC_ are shown in Table 7.

The experimental results show that when the aerosol concentration changes, the sensor can reach a stable value within about 10 s, that is, the sensor has a fast response speed for the aerosol concentration detection. As the concentration of aerosols increases, higher concentrations of aerosols require a longer time to achieve uniform distribution within the sensor detection space, which results in a relatively longer response and recovery time for the sensor. These results reveal the performance characteristics and response time variation patterns of sensors in dynamic aerosol detection.

#### 3.2.3. Performance Comparison with Other Aerosol Sensors

The comparison among the sensors proposed in this article and other types of sensors is shown in Table 8. It can be seen that the aerosol sensor proposed in this article has superior performance in many aspects.

## 4. Conclusions

The existing aerosol sensors have drawbacks such as large volume, insufficient sensitivity, poor anti-interference ability, and long response time. This paper proposes a four-sided micrometer column three-electrode ionization aerosol sensor based on field emission, field charging, and gas discharge theories. The experiment recorded the response characteristics of the sensor to 2.5 μm particle diameter aerosols in the concentration range of 0–6000 μg/m^3^ under pulse excitation conditions and a very high detection limit of 0.1 μg/m^3^ which is greatly superior to the existing technology. As the aerosol concentration increased, the collecting current showed a single-value decreasing trend. The pulse excitation parameter was optimized as 50% duty cycle and 80 kHz frequency. The sensors exhibit excellent performance in high sensitivity, small nonlinearity, high repeatability, fast response, and short recovery time, which is of great significance for the field of aerosol detection.

## Figures and Tables

**Table 1:** Dimension of the electrodes.

	Size of Electrodes	Size of Column Array	Number of Diffusion Holes	Diameter of Diffusion Holes	Size of the Groove
Cathode	7 mm × 8 mm	5 mm × 3 mm	9	0.6 mm	/
Extracting electrode	7 mm × 9 mm	5 mm × 3 mm	9	0.6 mm	/
Collecting electrode	7 mm × 10 mm	5 mm × 3 mm	/	/	2 mm × 2.4 mm; 200 μm depth

**Table 2:** Technical parameters of the power supply system.

Voltage/V	Current/mA	Frequency/kHz	Duty Cycle	Power Efficiency	Output Accuracy
0–100	0–200	1–10	0–90%	≥90%	≤1%

**Table 3:** Experimental conditions for the pulse excitation parameter optimization.

Parameters	Value
separation between electrodes/μm	30
extracting electrode voltage/V	70
collecting electrode voltage/V	1
pulse frequency/kHz	10/20/30/40/50/60/70/80/90
duty cycle/%	10/20/30/40/50/60/70/80/90

**Table 4:** External conditions of sensors.

Separation/μm	Temperature/°C	Pressure/kPa	Electrode Power Supply Conditions		
			Cathode	Extracting Electrode	Collecting Electrode
30	20	96	0 V	80 kHz, 50% duty cycle, 70 V	1 V

**Table 5:** Fitting results of the sensor response curve.

Fitting Curve Parameters	Goodness of Fit
*a*_0_ = 3681.197 A, *a*_1_ = −0.010 A/μg/m^3^, *a*_2_ = −6.160 × 10^−7^ A/(μg/m^3^)^2^, *a*_3_ = 1.061 × 10^−8^ A/(μg/m^3^)^2.5^, *a*_4_ = 12.253 A	0.991

**Table 6:** Static characteristic indicators of the sensor.

Output Full Range/nA	Output Zero Point/nA	Maximum Sensitivity/nA·μg/m^3^	Minimum Detection Limit/μg/m^3^
67.17	3693.45	−8.65 × 10^−2^	0.1
**Nonlinearity δ_L_**	**Hysteresis** **δ_H_**	**Repeatability** **δ_R_**	**Type A Uncertainty/μg/m^3^**
7.1%	2.1%	2.9%	3.21

**Table 7:** Dynamic response indicators of the sensor.

Concentration/μg/m^3^	t_1_/s	t_2_/s	t_3_/s	t_4_/s	*τ*_RES_/s	*τ*_REC_/s
1000	82	90	201	207	6	5
3000	104	113	239	246	7	6
6000	115	125	262	271	8	7

**Table 8:** Performance comparison of aerosol sensors.

Types	Range/μg/m^3^	Response Time/s	Size/mm	Error/%	Detection Limit/μg/m^3^
Sensor in this article	0–6000	<10	10 × 7 × 3	2.8	0.1
Winson ZPH04 particle sensor	0–500	<30	58.5 × 44.5 × 19.6	-	-
Honeywell HPM particle sensor	0–1000	-	43 × 36 × 23.7	<15	10.0
Honeywell vehicle aerosol sensor	200–5000	<12.2	66 × 37 × 36	<15	10.0
FDS 17 sensor	0–2000	<15	200 × 297 × 121	<15	5.0

## Data Availability

The original contributions presented in the study are included in the article, further inquiries can be directed to the corresponding author.
